# The structure and function of FUN14 domain-containing protein 1 and its contribution to cardioprotection by mediating mitophagy

**DOI:** 10.3389/fphar.2024.1389953

**Published:** 2024-05-17

**Authors:** Yuhu Lv, Zhengze Yu, Peiwen Zhang, Xiqian Zhang, Huarui Li, Ting Liang, Yanju Guo, Lin Cheng, Fenglin Peng

**Affiliations:** ^1^ College of Physical Education, Guangdong University of Education, Guangzhou, China; ^2^ Research Center for Adolescent Sports and Health Promotion of Guangdong Province, Guangzhou, China; ^3^ College of Physical Education and Health, Guangxi Normal University, Guilin, China; ^4^ College of Nursing and Rehabilitation, Xi an FanYi University, Xi’an, China

**Keywords:** FUN14 domain-containing protein 1, mitophagy, cardioprotection, mitochondria, exercise preconditioning

## Abstract

Cardiovascular disease (CVD) is a serious public health risk, and prevention and treatment efforts are urgently needed. Effective preventive and therapeutic programs for cardiovascular disease are still lacking, as the causes of CVD are varied and may be the result of a multifactorial combination. Mitophagy is a form of cell-selective autophagy, and there is increasing evidence that mitophagy is involved in cardioprotective processes. Recently, many studies have shown that FUN14 domain-containing protein 1 (FUNDC1) levels and phosphorylation status are highly associated with many diseases, including heart disease. Here, we review the structure and functions of FUNDC1 and the path-ways of its mediated mitophagy, and show that mitophagy can be effectively activated by dephosphorylation of Ser13 and Tyr18 sites, phosphorylation of Ser17 site and ubiquitination of Lys119 site in FUNDC1. By effectively activating or inhibiting excessive mitophagy, the quality of mitochondria can be effectively controlled. The main reason is that, on the one hand, improper clearance of mitochondria and accumulation of damaged mitochondria are avoided, and on the other hand, excessive mitophagy causing apoptosis is avoided, both serving to protect the heart. In addition, we explore the possible mechanisms by which FUNDC1-mediated mitophagy is involved in exercise preconditioning (EP) for cardioprotection. Finally, we also point out unresolved issues in FUNDC1 and its mediated mitophagy and give directions where further research may be needed.

## 1 Introduction

Myocardial infarction (MI) is an acute coronary syndrome in cardiovascular disease (CVD) that progresses from myocardial cell death to myocardial injury and cardiac dysfunction as a progressive factor in heart failure (HF) and death (Yellon and Hausenloy, 2007; Li et al., 2022a; Lv et al., 2022). Myocardial reperfusion is the primary strategy for reducing the size of MI. However, it relieves symptoms while not addressing myocardial cell death and loss, and reperfusion itself induces ischemia/reperfusion (I/R) injury (IRI), which has been one of the significant challenges in the field. At the same time, mitochondrial dysfunction, oxidative stress, calcium overload, pH paradox, and inflammation are all potential factors in the pathogenesis of IRI (Mitrega et al., 2016; Bi et al., 2018; Heusch, 2020; Mao et al., 2021; Xing et al., 2022). As research progresses, more and more pieces of evidence point to mitochondria, which bear the brunt when ischemic damage occurs in cardiomyocytes (Li et al., 2019; Livingston et al., 2019; Pecoraro et al., 2019).

The emergence of mitochondria was a turning point in the evolution of species. Although theories are numerous and varied, they all point to the endosymbiotic theory that mitochondria originated from bacteria, which suggests that bacteria engulfed by eukaryotes evolved and adapted over time to form mitochondria to adapt to the highly oxygenated environment that occurred in the atmosphere (Szklarczyk and Huynen, 2010). The mitochondria are highly dynamic organelles that form a mitochondrial network by continuously fusing, fissioning, and moving along the cytoskeleton (Tagaya and Arasaki, 2017). It was demonstrated that mitochondria are not only random sites of oxidative and calcium-mediated damage but also trigger mitochondrial remodeling and activation of cellular responses and regulate the balance between cell death and recovery (Lesnefsky et al., 2017). Mitochondria in eukaryotic cells are involved in energy production, thermoregulation, metabolite biosynthesis, calcium signaling, redox homeostasis, inflammatory response, and apoptosis through oxidative phosphorylation (OXPHOS) and the electron respiratory chain, and their nature and function are not identical in different organs, tissues or cells, and this multifunctionality enables adapting cells to changes in various environments and stimuli (Ong et al., 2013; Mohsin et al., 2021; Zhang, 2021). Senescent and damaged mitochondria produce large amounts of reactive oxygen species (ROS), which can induce oxidative stress damage and even apoptosis, therefore needing to be removed promptly, a role played by selective mitochondrial autophagy (Ji et al., 2021). During pathological conditions, noxious stimuli may inhibit mitophagy or increase its impaired amount beyond its ability to selectively regulate autophagy, which in turn leads to the accumulation of damaged mitochondria and the release of cytochrome C and a series of pro-apoptotic factors, thus inducing oxidative stress or mitochondrial-dependent cell death (Ji et al., 2021).

Autophagy is an evolutionarily conserved degradation process, and mitophagy is a type of selective autophagy, which is the process of removing damaged or dysfunctional mitochondria by selective autophagy, including typical and atypical mitophagy (Ray and Mukherjee, 2021). Typical mitochondrial autophagic pathways include PINK1/PARKIN, BCL2 interacting protein 3 (BNIP3)/pro-apoptotic protein Nip3 (NIX), and the FUN14 domain-containing protein 1 (FUNDC1) -mediated mitophagy (Lampert et al., 2019; Teresak et al., 2022; Ma et al., 2024). Atypical mitochondrial autophagic pathways include autophagy and Beclin 1 regulator 1 (AMBRA1), prohibitin-2 (PHB-2), nucleotide-binding (NB) domain -and leucine-rich repeat (LRR)-containing proteins (NLR) X1 (NLRX1), lipids, cardiolipids (especially diphosphatidylglycerol CL), ceramides, BCL2L13, FKBP8, Rab-mediated mitophagy and micro-mitophagy (When mitochondria are mildly damaged or present only in small areas, mitochondrial-derived vesicles (MDVs) containing specific proteins that can be degraded after transport to lysosomes with the assistance of PINK1) (McLelland et al., 2014; Bhujabal et al., 2017; Lim and Lim, 2017; Saito et al., 2019; Belousov et al., 2021; Li et al., 2021b; Ma et al., 2021; Ray and Mukherjee, 2021; Choubey et al., 2022). In this review, we focus on the role of FUNDC1 and its mediated mitophagy in cardioprotection. In addition, we explore the possible mechanisms by which FUNDC1-mediated mitophagy is involved in exercise preconditioning (EP) for cardioprotection. This work contributes to the development of new strategies for the treatment of many diseases, especially CVD.

## 2 Mitochondria and mitophagy and their roles in the heart

Studies have shown that cardiomyocytes contain a large number of mitochondria, equivalent to 30%–40% of their total volume, and that approximately 6 kg of adenosine triphosphate (ATP) consumed daily under physiological conditions in the adult heart is produced through mitochondria (equivalent to approximately 90% of total cardiac energy consumption); mitochondria face challenges in performing their cellular duties such as oxidative stress, altered protein relationships (protein import, folding and degradation) and mitochondrial DNA damage, they respond to these challenges through robust quality control mechanisms, including post-translational modification of mitochondrial proteins, mitochondrial dynamics, antioxidant defence, biogenesis and mitophagy, which are critical to mitochondrial and even cellular homeostasis under physiological or pathological conditions; failure of quality control will result in damage to mitochondria, which in turn will cause altered substrate utilisation, failure of quality control will result in impaired mitochondria, leading to altered substrate utilisation, OXPHOS impairment, ATP deficiency, excessive ROS accumulation, impaired metabolic signalling and inflammation (Deng et al., 2017; Wang et al., 2017; Fan et al., 2020; Li et al., 2022a; Choubey et al., 2022). The accumulation of dysfunctional mitochondria is detrimental to cells and organisms and is a typical feature of the etiology of related diseases, therefore maintaining a healthy mitochondrial pool (compensating for mitochondrial function through mitochondrial biogenesis, fusion, and fission, as well as degrading damaged mitochondria through mitophagy) is necessary for cell function and survival, and mitophagy is generally considered to play a crucial role in this (Vigie and Camougrand, 2017; Yoo and Jung, 2018; Sun et al., 2021; Zhang, 2021). The term “mitophagy” was first used in 1998, but it is generally accepted that mitophagy was first discovered in 1914 and confirmed by the observation of mitochondrial fragments in the electron microscope in 1962 (Lewis and Lewis, 1914; Ashford and Porter, 1962; Scott and Klionsky, 1998; Wang et al., 2019a). The mechanism mainly involves depolarization and loss of the outer membrane potential upon mitochondrial damage by external stimulation, followed by autophagosome engulfment of the mitochondria to form mitochondrial autophagosomes and subsequent degradation by lysosomal binding.

Mitochondria exhibit structural and functional abnormalities in CVD, such as cardiac hypertrophy, heart failure, and ischaemic cardiomyopathy (Pecoraro et al., 2019). The critical role of mitochondria in cardiac function also makes them an essential target for IRI, and I/R induces mitochondrial cristae damage, abnormal membrane potential, and excessive opening of the permeability transition pore (PTP), leading to mitochondrial dysfunction. The damaged mitochondria produce ten times more ROS than normal mitochondria, exacerbating mitochondrial dysfunction and causing further damage, and so on, in a vicious cycle (Tombo et al., 2020; Luan et al., 2021; Mao et al., 2021). Excessive damage to mitochondria also triggers the cell death pathway, which ultimately leads to tissue breakdown (Goldenthal, 2016; Li et al., 2022a; Pei et al., 2024). Mitochondrial dysfunction is considered to be the most critical molecular mechanism responsible for myocardial IRI and heart disease and is also closely linked to functional mitophagy, which further constitutes a developmental program and occurs in high crosstalk with apoptosis (Cung et al., 2015; Hamacher-Brady and Brady, 2016; Aghaei et al., 2019; Luan et al., 2021).

Mitochondria are the hub of the cellular metabolic network and an essential organelle for the regulation of oxidative stress, autophagy, and apoptosis, whose quality control is mainly dependent on the stability of mitophagy, and a growing body of evidence suggests that mitochondrial quality control in cardiomyocytes has a critical role in improving cardiac function, rescue dying cardiomyocytes and prevent the deterioration of CVD in response to external environmental stress, and where functional mitophagy is essential to maintain their quality and quantity, allowing rapid clearance of damaged mitochondria before they can cause damage to the cell (Yang et al., 2019; Fan et al., 2020; Zhang et al., 2021a; Li et al., 2021c; Ji et al., 2021; Zhang, 2021; Choubey et al., 2022). It has been well demonstrated that autophagy, particularly mitophagy, acts as an agent in the protective effect of ischemic preconditioning (IPC) (Livingston et al., 2019). The process of mitophagy needs to be confined to senescent or dysfunctional mitochondria and maintained at an equilibrium level, the disruption of which inevitably leads to cardiomyocyte damage and dysfunction (Li et al., 2019). Therefore, scholars believe that proper mitophagy can protect the myocardium from IRI (Xiao et al., 2020; Huang et al., 2021; Ji et al., 2022). Autophagy targeting mitochondria, so-called mitophagy, has also been suggested as a possible promising strategy to protect the myocardium from IRI (Cung et al., 2015; Aghaei et al., 2019; Yu and Miyamoto, 2021). Due to its great potential utility, mitophagy is a current burning topic in the molecular mechanisms of organelle-specific autophagy.

## 3 Structure and function of FUNDC1 and its mediated pathways of mitophagy

### 3.1 Structure and function of FUNDC1

**FIGURE 1:**
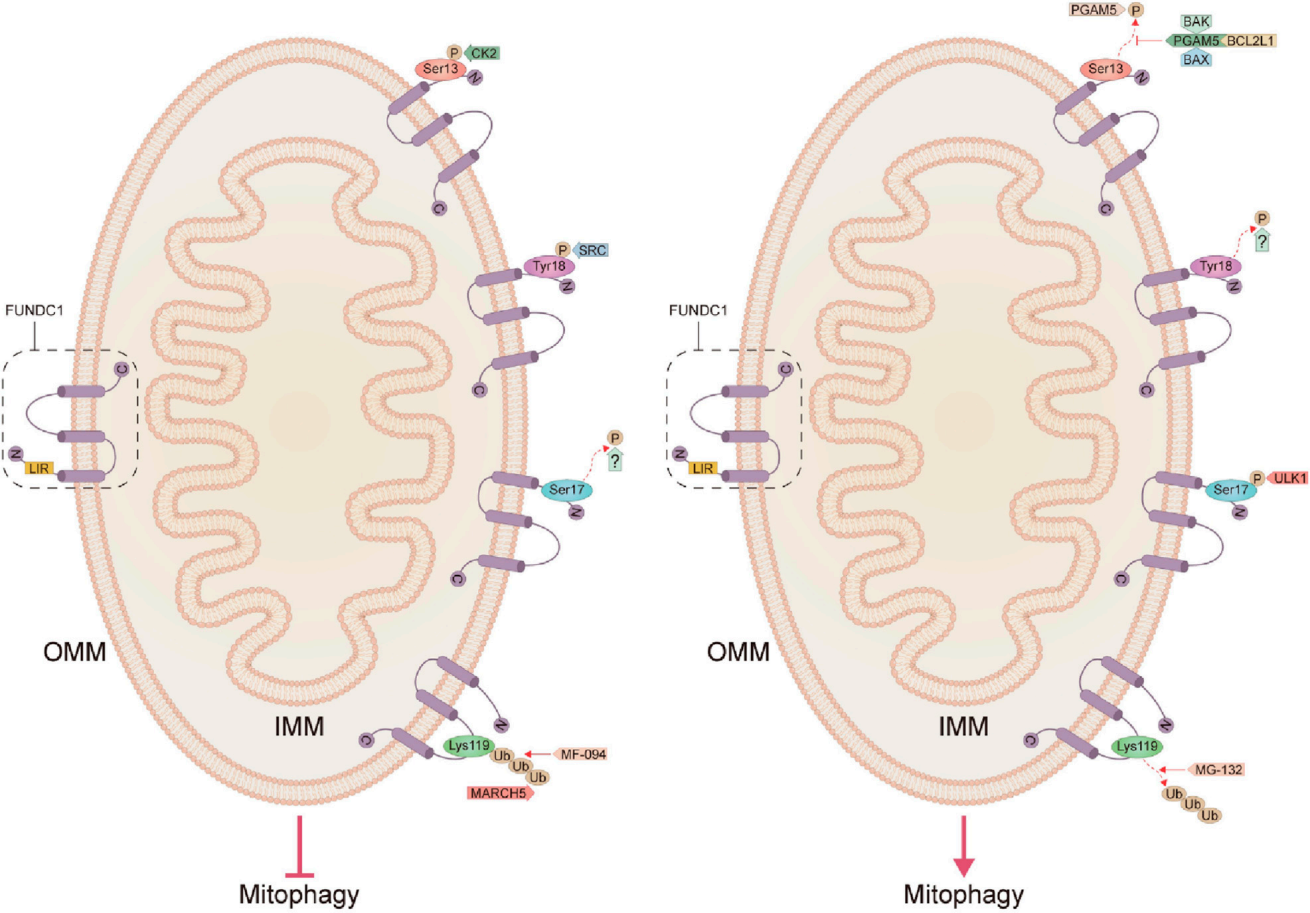
Structure of FUNDC1 and the pathways that inhibit and activate mitophagy. C, C-terminal; N, N-terminal; IMM, inner mitochondrial membrane; OMM, outer mitochondrial membrane; LIR, LC3 interaction region; BCL2L1, BCL2-like1; CK2, Casein kinase 2; FUNDC1, FUN14 domain-containing protein 1; IMM, inner mitochondrial membrane; OMM, outer mitochondrial membrane; MARCH5, mitochondrial E3 ubiquitin ligase membrane-associated RING-CH5; PGAM5, phosphoglycerate mutase 5; SRC, Src proto-oncogene kinase; Ub, ubiquitination; ULK1, unc-51-like autophagy-activated kinase 1.

FUNDC1 is an outer mitochondrial membrane protein with a conserved sequence ranging from *Drosophila melanogaster* to *Homo sapiens*, discovered and named by the State Key Laboratory of Membrane Biology, Institute of Zoology, Chinese Academy of Sciences, in 2012 (Liu et al., 2012). The human FUNDC1 protein contains 155 amino acids and contains three transmembrane fragments, with the C-terminal extending into the membrane gap and the N-terminal (AAs1-50) exposed in the cytoplasm, and is widely expressed, especially in the heart (Figure 1) (Liu et al., 2012). It was shown that autophagic receptors always target cargoes to be degraded (damaged organelles, protein aggregates, or invading pathogens) with microtubule-associated protein 1 light chain 3 (MAP1LC3) members (including MAP1LC3A, MAP1LC3B, MAP1LC3B2, and MAP1LC3C) or homologs (GABARAP, GABARAPL1, and GABARAPL2) interact through the LC3 interaction region (LIR), tethering them to the autophagosomal membrane, where typical LIRs include a (W/F/Y)XX (L/I/V) core pattern interacting with MAP1LC3, and two hydrophobic pockets of LIR docking sites in the homologs anchored to the autophagosomal membrane (Zhang et al., 2021c). Related studies tested 30 LIRs, 12 of which (40%) were selective for GABARAP, but only one LIR motif (Y18-E19-V20-L21) FUNDC1 preferentially interacted with LC3, and mutations in the LIR motif would impair its interaction with LC3 and subsequently impede the process of mitophagy (Liu et al., 2012; Kuang et al., 2016; Rogov et al., 2017; Zhang, 2021; Liu et al., 2022).

In addition to inducing mitophagy, FUNDC1 has a critical role in the maintenance of normal mitochondrial morphology and function in cardiomyocytes, interacting with inositol-1,4,5-triphosphate receptor 2 (IP3R2) in modulating Ca^2+^ release from the endoplasmic reticulum (ER) into the mitochondria and cytoplasm, and disruption of its interaction decreases Ca^2+^ levels in the mitochondria and cytoplasm, triggering abnormal mitochondrial fission, mitochondrial dysfunction, cardiac dysfunction, and HF (Wu et al., 2017a). Under basal conditions, FUNDC1 binds to the mitochondria-associated ER membranes (MAMs) protein calnexin (CNX), and with mitophagy, the FUNDC1/CNX association decreases, and the FUNDC1-exposed cytoplasmic loop interacts with dynamin-related protein 1 (DRP1), which is recruited to MAMs, promoting fission of mitochondria and preventing them from experiencing hypoxic stress (Chen et al., 2016; Wu et al., 2016; Chai et al., 2021). FUNDC1 also has several other functions, such as activating the unfolded protein response (UPR) of mitochondria (UPR_mt_) for maintaining mitochondrial quality control, participating in analgesia with hyperbaric oxygen, promoting adaptive thermogenesis, and regulating body metabolism (Wu et al., 2019a; Liu et al., 2020; Liu et al., 2021b; Ji et al., 2022). It was demonstrated that endogenous UPR_mt_ and mitophagy may be mildly activated in response to myocardial stress and that both act together to maintain mitochondrial performance and cardiac function, whereas exogenous UPR_mt_ is a downstream signal of mitophagy and serves as a compensatory role in maintaining mitochondrial homeostasis in the presence of mitophagy inhibition, and mitophagy coordinates UPR_mt_ to attenuate inflammation-mediated myocardial injury (Wang et al., 2021).

### 3.2 FUNDC1-mediated pathways of mitophagy

FUNDC1 is expressed at a high level in the myocardium, providing support for its critical role in cardiac function (Wu and Zou, 2019). The FUNDC1, a mitophagy receptor, occupies a critical role in mitochondrial quality control by regulating mitophagy and is also strongly associated with the development of some CVD (Li et al., 2021a; Liu et al., 2021a). It was shown that FUNDC1-mediated mitophagy is activated primarily in cardiomyocytes and is essential for mitochondrial network remodeling in the process of cardiac progenitor cell homeostasis and differentiation (Lampert et al., 2019; Zhang, 2021). FUNDC1 deficiency aggravated doxorubicin-induced cardiac dysfunction, mitochondrial damage, and cardiomyocyte PANoptosis (Bi et al., 2022). The mechanism of transcriptional regulation of mitophagy remains unclear (Li et al., 2023). It was shown that miR137 mimics introduced under hypoxic conditions could inhibit mitophagy by targeting FUNDC1 (Hu et al., 2020). Overexpression of miR-137 triggers a series of molecular alterations such as Nix, LC3B, and FUNDC1, leading to fragmentation and densification of mitochondrial ultrastructure, and ultimately leading to aberrant mitophagy (Khadimallah et al., 2021). miR-137 may reduce FUDC1-LC3 by inhibiting the overexpression of fundc1 (CDS+3UTR) rather than fundc1 (CDS), and thus inhibiting FUDC1-LC3 interaction, which in turn inhibits mitophagy (Li et al., 2014). A different view also pointed out that no downregulation of FUNDC1 was found in miR-137-transfected pancreatic cancer cells PANC-1, but downregulated the mRNA and protein levels of endogenous ATG5 (Wang et al., 2019b). It suggests that miR137 may have multiple functions. However, the post-translational regulation of FUNDC1 has been more clearly defined.

#### 3.2.1 The dephosphorylation pathway of Ser13 on the LIR motif

The phosphorylation status of FUNDC1 significantly affects its mediated mitophagy, and reversible phosphorylation modification of the mitophagy receptors might be the molecular switch for selective mitophagy (Lv et al., 2017). Casein kinase 2 (CK2) is a constitutive serine/threonine kinase that inhibits mitophagy by inducing phosphorylation of Ser13 on the LIR motif in FUNDC1 under normal conditions while, in contrast, activating mitophagy when dephosphorylated at Ser13 (Kuang et al., 2016; Zhou et al., 2018). Disruption of the two catalytic subunits α1 and α2 of CK2 under physiological conditions significantly abolished its ability to phosphorylate Ser13 on FUNDC1 and enhanced FUNDC1-mediated mitophagy, confirming that CK2 is the protein kinase with responsibility for Ser13 site phosphorylation of FUNDC1 (Zhang, 2021). The FUNDC1-mediated activity of mitophagy is also fine-tuned by the anti-apoptotic protein BCL2-like1 (BCL2L1, also known as BCL-XL) through the regulation of phosphoglycerate mutase 5 (PGAM5) activity, which is inhibited during steady-state by the BCL2 homology 3 domain (BH3) in a mechanism based on the formation of a complex of BCL-XL in a non-phosphorylated form with the pro-apoptotic proteins BAX, BAK, and PGAM5, which then inhibits the dephosphorylation of FUNDC1 at the Ser13 site and consequently the subsequent mitophagy (Wu et al., 2014a). The direct interaction of BCL-XL with PGAM5 also regulates apoptosis, as when BCL-XL in the complex is released, PGAM5 reactivates its anti-apoptotic function by dephosphorylating BCL-XL in its dimeric state (Ma et al., 2020). In contrast, PGAM5 oligomerization due to mild oxidative stress eliminates its ability to bind to BCL-XL while retaining its ability to dephosphorylate FUNDC1, thus acting to activate mitochondrial division and mitophagy for cell survival (Ma et al., 2020). It has been shown that both hypoxia and carbonyl cyanide 4-(trifluoromethoxy) phenylhydrazone (FCCP) attenuate the interaction of BCL-XL with PGAM5 and release PGAM5, which in turn promotes the dephosphorylation of FUNDC1 and activates mitophagy (Wu et al., 2014a). CK2 counteracts the effects of PGAM5 under basal conditions by reversible phosphorylation of the Ser13 site on FUNDC1 to prevent autophagic clearance of mitochondria (Figure 1) (Chen et al., 2014).

At the initiation of mitophagy, PGAM5 can be cleaved by a rhombic protease called PARL (which degrades PINK1 in healthy cells), which later promotes the phagocytosis of damaged mitochondria via dephosphorylation of the mitophagy receptor FUNDC1 by autophagosomes (Sugo et al., 2018). Recently, syntaxin17 (STX17) located in MAMs was also demonstrated to be critical for PGAM5 dephosphorylation of FUNDC1 during mitophagy, and interestingly, like NIX/BNIP3L and BNIP3, FUNDC1 may also be involved in PINK1-PARKIN-dependent mitophagy, as FUNDC1 deletion inhibits carbonyl cyanide-m-chlorophenylhydrazone (CCCP)-induced mitochondrial clearance, but also prevents complete PARKIN coverage of mitochondria and the mitochondrial aggregation observed after CCCP treatment (Sugo et al., 2018). The process of mitophagy induced by BINP3L, FUNDC1, and PARKIN probably does not arise independently, as these factors promote their recruitment not only through a positive feedback loop but also through the recruitment of other factors that promote mitophagy (Zhang et al., 2021d). However, a different view has also been proposed that FUNDC1 regulates mitophagy in rotenone-treated SH-SY5Y cells in an independent manner from the PINK1/PARKIN-dependent pathway (Park and Koh, 2020). These findings above suggest the possibility that different pathways of mitophagy may coordinate with each other, so it could be very interesting to further investigate whether BNIP3 or NIX/BINP3L-mediated mitophagy can compensate for the depletion of FUNDC1 (Choubey et al., 2022).

#### 3.2.2 The dephosphorylation pathway of Tyr18 on the LIR motif

LC3 preferentially interacts with dephosphorylated FUNDC1, probably because phosphorylated FUNDC1 conflicts with the hydrophobic pocket of LC3, which in turn eliminates the affinity of FUNDC1 to bind LC3 (Kuang et al., 2016). Normally, Src proto-oncogene kinase (SRC) could induce phosphorylation of Tyr18 on the LIR motif in FUNDC1 and attenuate the interaction with LC3, thereby inhibiting mitophagy (Kuang et al., 2016; Zhou et al., 2017b). Unlike other LIR-containing autophagy receptors where dephosphorylation normally inhibits binding affinity to LC3 and suppresses autophagy, dephosphorylation of the LIR in FUNDC1 promotes its LC3-dependent binding, which in turn enhances hypoxia-induced mitophagy (Zhang, 2021). It was noted that inhibition of either CK2 or SRC could not fully activate FUNDC1-mediated mitophagy, but synergistic inhibition of both kinases significantly activated mitophagy, while further structural and functional analyses indicated that reversible phosphorylation modification of Tyr18 on LIR of FUNDC1 is a novel molecular switch for its mediated mitophagy and that the phosphorylation state of Tyr18 in the LIR motif characterized by Y (18)EVL (21) characterizes the phosphorylation state of Tyr18 in the LIR motif plays a central role in regulating the affinity of FUNDC1 to bind LC3 and controls FUNDC1-mediated mitophagy activity, whereas the phosphorylation state of Ser13 in the LIR of FUNDC1 does not significantly alter its affinity to bind LC3, and therefore FUNDC1 phosphorylation at Ser13 dephosphorylation may act as an adjunct to promote FUNDC1-mediated mitophagy (Zhang, 2021). So far nothing has been found to mediate the dephosphorylation of the LIR motif Tyr18 in FUNDC1, which seems to be a gap, and future exploration in this direction will be fascinating (Figure 1).

#### 3.2.3 Phosphorylation pathway of Ser17 on the LIR motif

It was shown that in addition to dephosphorylation of Ser13 and Tyr18 sites on the LIR motif in FUNDC1 could activate mitophagy, phosphorylation of Ser17 site on FUNDC1 could also promote mitophagy (Kuang et al., 2016). By forming a hydrogen bond between the Arg10 side-chain of LC3B and the Ser13 side-chain and the carbonyl group of the FUNDC1 backbone, the phosphorylation of this residue prevented the LC3B-FUNDC1 interaction through steric effects (Kuang et al., 2016; Lv et al., 2017). In response to reduced ATP, adenosine monophosphate-activated protein kinase (AMPK) phosphorylates the Ser313, Ser555, and Ser777 sites in unc-51-like autophagy-activated kinase 1 (ULK1), and phosphorylated ULK1 promotes phosphorylation of the Ser17 site on the LIR motif in FUNDC1, and consequently, selective mitochondrial incorporation into the LC3 or GABARAP-bound isolation membrane, followed by cleared by autolysosomes (Wu et al., 2014b; Liu et al., 2022; Turkieh et al., 2022). Which phosphatase is currently involved in the dephosphorylation of FUNDC1 at the Ser17 site is still unknown, and enhanced exploration of this aspect is important for future studies (Figure 1).

#### 3.2.4 The ubiquitination pathway at the Lys119 site

In addition to phosphorylation and dephosphorylation, mitochondrial E3 ubiquitin ligase membrane-associated RING-CH5 (MARCH5/MITOL)-mediated ubiquitination of FUNDC1 at Lys119 could also inhibit initial hypoxia-induced mitophagy through degradation of the proteasome of FUNDC1 (Chen et al., 2017a; Wu et al., 2017b; Chen et al., 2023). Under hypoxic stress, MARCH5 degrades excessive FUNDC1 to fine-tune hypoxia-induced mitophagy, while ablation of MARCH5 results in an exaggerated phenotype of mitophagy and FUNDC1 accumulation, whereby the mechanism is that hypoxic damage enhances the Lys119 site of MARCH5 ubiquitinating FUNDC1 for subsequent degradation, which avoids inappropriate mitochondrial clearance, and severe hypoxia-induced dephosphorylation of FUNDC1 increases the flux of mitophagy, and therefore the mechanism regulating the MARCH5/FUNDC1 axis may be negative feedback, avoiding inappropriate clearance of intact mitochondria (Chen et al., 2017a; Chen et al., 2017b). The level of ubiquitination of FUNDC1 can be both inhibited by the proteasome inhibitor MG-132 and activated by the proteasome activator MF-094 (Chen et al., 2022a). The increased FUNDC1 ubiquitination levels inhibited mitophagy and changes in mitochondrial membrane potential (Δψm) in hypoxic trophoblast cells, thereby reducing oxidative damage, which again demonstrates that FUNDC1 ubiquitination of Lys119 in FUNDC1 can regulate mitophagy (Figure 1) (Chen et al., 2022a).

In summary, the mechanism by which FUNDC1 mediates mitophagy is that when dephosphorylation of the Ser13/Tyr18 site on the LIR motif or phosphorylation of the Ser17 site on the LIR motif or deubiquitination of Lys119 in FUNDC1 can result in mitochondria being selectively admitted to LC3-bound or GABARAP-bound detachment membranes and subsequently cleared by autolysosomes. With the progression of mitophagy, the association of FUNDC1 with calnexin is weakened and the exposed FUNDC1 cytoplasmic loop interacts with DRP1, which is consequently recruited to the mitochondria-associated ER-membranes, and mitochondrial fission ensues (Chen et al., 2016; Wu et al., 2016). On the contrary, the affinity of FUNDC1 for LC3 and GABARAP was hindered, which in turn inhibited mitophagy.

## 4 Roles of FUNDC1-mediated mitophagy in cardioprotection

### 4.1 Activation of FUNDC1-mediated mitophagy

It was shown that, on the one hand, 45 min of ischemia significantly reduced the inhibitory phosphorylation by the SRC at the Tyr18 site of FUNDC1, and on the other hand, the interaction of PGAM5 with the Ser13 site of FUNDC1 during hypoxia dephosphorylated FUNDC1, which combined to enhance the interaction of FUNDC1 with LC3 and thus activate mitophagy (Chen et al., 2014). In response to I/R stress, cardiac structure, and function could be maintained by reducing the effects of CK2 and upregulating FUNDC1-dependent mitophagy, while an increase in CK2 is induced after cardiac IRI (Zhou et al., 2018). FUNDC1-mediated mitophagy is also regulated by other factors, such as activation of the AMPKα1/ULK1/FUNDC1/mitophagy pathway can attenuate cardiac microvascular IRI, and a study on the Danqi pill also showed that Danqi pill could enhance FUNDC1-mediated mitophagy by modulating ULK1 and PGAM5 to protect HF after acute MI (Wang et al., 2022b; Cai et al., 2022). Similarly, a study on alpha-lipoic acid showed that it could protect the heart from pressure overload-induced HF by activating FUNDC1-mediated mitophagy (Li et al., 2020). FUNDC1-mediated mitophagy may also act synergistically with other responses, such as BAX inhibitor-1 (BI-1) can ameliorate myocardial injury in type 3 cardiorenal syndrome by activating UPR_mt_ and FUNDC1-associated mitophagy (Wang et al., 2022a). It has also been shown that mammalian sterile 20-like kinase 1 (MST1) can promote cardiac IRI by inhibiting FUNDC1-dependent mitophagy by inhibiting the mitogen-activated protein kinase (MAPK)/ERK-CREB pathway, while the genetic ablation of MST1 can reverse this FUNDC1-involved mitophagy, thereby eliminating mitochondrial damage and cardiomyocyte death and ultimately preventing IRI in the heart (Yu et al., 2019; Shang et al., 2022). It indicates that MST1 may be one of the upstream regulators of FUNDC1-mediated mitophagy. A cellular-level study showed that irisin in lipopolysaccharide-stimulated H9c2 cardiomyocytes could abrogate mitochondrial dysfunction, oxidative stress, and apoptosis through FUNDC1-related mitophagy, and thus act as a treatment for infectious cardiomyopathy (Jiang et al., 2021b). These studies provide definite evidence for modulating mitophagy and provide a reference for drug development.

### 4.2 Inhibition of FUNDC1-mediated mitophagy

After IRI, progressively increased CK2 in the heart inhibits protective mitophagy by post-transcriptional inactivation of FUNDC1, which in turn promotes mitochondrial apoptosis in cardiomyocytes and the progression of myocardial IRI (Zhou et al., 2018). Utilizing Beclin1^+/−^, FUNDC1 gene knockout, and FUNDC1 transgenic mice combined with starvation and MI model, it was found that after MI, the FUNDC1 knockout group caused more severe mitochondrial and cardiac dysregulation than the Beclin1^+/−^ group, suggesting that mitophagy but not macroautophagy promotes cardioprotection primarily by regulating mitochondrial function (Xu et al., 2022). The same results were seen with baseline genetic ablation of FUNDC1, as evidenced by reduced early to late ventricular filling velocity, prolonged left ventricular isovolumic diastole, and reduced ejection fraction in mice and these phenotypic alterations suggest that FUNDC1 knockout mice are susceptible to HF (Wu et al., 2017a; Zhu et al., 2021). It has also been shown that serine/threonine-protein kinase 3 (RIPK3) can directly bind to FUNDC1 and inhibit mitophagy (Zhou et al., 2017b). Deletion of RIPK3 in cardiomyocytes or microvascular endothelial cells in IRI reduces cardiomyocyte apoptosis, ROS production, and mitochondrial fragmentation and activates mitophagy (Zhou et al., 2017b). Deletion of RIPK3 *in vivo* also improved cardiac function, whereas overexpression of RIPK3 exacerbated cardiac dysfunction by inhibiting FUNDC1-mediated mitophagy (Zhou et al., 2017b).

In contrast to the view that activation of mitophagy facilitates cardiac function, some investigators have suggested that excessive mitochondrial elimination induced by I/R increases the death of cardiomyocytes (Lesnefsky et al., 2017; Ji et al., 2021). A study showed that melatonin could effectively inhibit platelet activation by restoring peroxisome proliferator-activated receptor gamma (PPARγ) levels in platelets, thereby blocking FUNDC1-mediated mitophagy to protect the heart from IRI (Zhou et al., 2017a). The effect of moxibustion in relieving chronic HF may also be related to the inhibition of FUNDC1-mediated mitophagy (Xia et al., 2022). The removal of FUNDC1-dependent mitophagy could render the myocardium resistant to paraquat-induced contractile dysfunction (Peng et al., 2022). A similar result was reported in the study of electroacupuncture preconditioning, which attenuated myocardial IRI by inhibiting mTORC1/ULK1/FUNDC1 pathway-mediated mitophagy (Xiao et al., 2020). This was also supported by another study, which showed that I/R increased the expression of FUNDC1 and LC3II/LC3I ratio and decreased the expression of p-mTORC1/mTORC1, while electroacupuncture preconditioning reversed this trend and suggested that electroacupuncture preconditioning could reduce brain deficit IRI by inhibiting mitophagy (Mao et al., 2020).

## 5 EP/exercise and hypoxia modulate FUNDC1-mediated mitophagy in cardioprotection

### 5.1 Exercise/EP

IPC has previously been clinically demonstrated to exhibit cardioprotective effects, and EP has been both experimentally and clinically demonstrated to exhibit cardioprotective effects due to its similar effects to IPC (Quindry and Hamilton, 2013). EP can be divided into early EP (EEP) and late EP (LEP), both of which have a myocardial protective effect (Thijssen et al., 2018). Appropriate exercise has a variety of functions, including but not limited to enhancing memory, improving cognition, suppressing obesity, and reducing inflammation (Chen et al., 2021; De Miguel et al., 2021; Li et al., 2022b). Exercise is an effective instrument for the prevention, intervention, and treatment of metabolic diseases, and although there are many possible mechanisms by which EP mediates cardioprotection, it appears that mitochondria exert an essential role in some of these mechanisms, with growing evidence supporting the protection induced by exercise mediated by autophagy, mitophagy, and mitochondrial biogenesis (Roberts and Markby, 2021; Li et al., 2022b; Ma et al., 2023). Exercise also induces FUNDC1-mediated mitophagy, removes damaged mitochondria, reverses mitochondrial dysfunction, stimulates mitochondrial biogenesis, and has a significant effect on mitochondrial fission and fusion via the AMPK-ULK1 pathway (Laker et al., 2014; Moreira et al., 2017; Gao et al., 2020; Yu et al., 2020; Memme et al., 2021). An acute exercise study concluded that PINK1 was not associated with skeletal muscle exercise-induced mitophagy because no significant PINK1 was present in mitochondria separated from skeletal muscle at any time points after acute exercise, but there was unambiguous evidence of stable PINK1 in mitochondria after treatment of HeLa cells with the uncoupling agent CCCP (Drake et al., 2019). This may be related to FUNDC1-mediated mitophagy, but whether similar results are found in the heart requires further studies to confirm. One study suggests that FUNDC1-mediated mitophagy may be influenced by the type of exercise (Laker et al., 2017).

Exercise may have a cardioprotective role by modifying FUNDC1 to regulate MAMs. FUNDC1, which tethers IP3R2, regulates both Ca^2+^ homeostasis and MAMs, exerts a crucial role in mitochondrial quality control, and is closely associated with the development of multiple CVD (Liu et al., 2021a). Research in septic mice found that upregulation of FUNDC1-dependent formation of MAMs promotes cardiac dysfunction (Jiang et al., 2021a). Conversely, FUNDC1 deficiency exacerbated high-fat diet-induced remodeling of the heart, mitochondrial aberrations, cell death, elevated IP3R3, and Ca^2+^ overload in FUNDC1^−/−^ mice (Ren et al., 2020). Diabetes may induce the formation of MAMs by downregulating AMPK, and activation of AMPK may play a part in ameliorating diabetic cardiomyopathy by downregulating FUNDC1 and FUNDC1-associated MAMs (Wu et al., 2019b; Chen Y. et al., 2022). Lack of FUNDC1 decreases IP3R2 and Ca^2+^ levels in mitochondria and cytoplasm and reduces mitochondrial dysfunction, cardiac dysfunction, and HF by inhibiting Ca^2+^-sensitive CREB-mediated fission 1(FIS1) expression (Wu et al., 2017a; Zhang et al., 2018). Although the role of MAMs in FUNDC1-mediated mitophagy needs to be further investigated, the available evidence demonstrates that MAMs provide a platform for FUNDC1 to exert its bio functions (Yang et al., 2020).

Although the merits of autophagy on myocardial ischemic injury remain controversial, the protective effect of exercise on the myocardium is clear, and modulation of mitophagy by exercise may be a promising cardioprotective strategy (Gedik et al., 2014). Although some exercise regimens are known to promote the protective effects of mitophagy in the heart, further research is still needed to determine the precise mechanisms of interaction between functional mitophagy and physical activity, to analyze and identify the possible factors influencing these mechanisms, and to investigate exercise modalities that are both convenient and induce mitochondrial adaptation, which would also greatly help to address exercise compliance (Moreira et al., 2017; Memme et al., 2021).

### 5.2 Hypoxia

The promotion of FUNDC1-mediated mitophagy under hypoxic conditions can play a role in protecting cardiomyocytes (Li et al., 2018). Hypoxia activates mitophagy through the phosphorylation of ULK1 at the Ser555 site induced by AMPK, while the inhibition or knockdown of the AMPK gene inhibits mitophagy by preventing the translocation of ULK1 (Tian et al., 2015; Ponneri Babuharisankar et al., 2023). Mechanistically, the ULK1 is upregulated and translocated to the damaged mitochondria during hypoxia, which proceeds to interact with FUNDC1 and phosphorylate FUNDC1 at the Ser17 site, enhancing the binding of FUNDC1 to LC3 and promoting mitophagy (Wu et al., 2014b). Mitophagy plays a critical role in the reduction of IRI by hypoxic preconditioning, and the decrease in oxygen levels induced by hypoxia or ischemia increases mitophagy by decreasing FUNDC1 phosphorylated at the Tyr18 site, induced by mitochondrial degradation (Zhang et al., 2016). In contrast, it has been suggested that inhibition of mitophagy may protect cells from hypoxia-induced damage, such that inhibition of mitophagy by introducing miR-137 mimics targeting FUNDC1 under hypoxic conditions promotes mitochondrial mass, ATP synthesis, and mitochondrial transcriptional activity, and a similar effect was obtained by specific siRNA knockdown of FUNDC1 (Hu et al., 2020). It was recently shown that miR-130a can regulate FUNDC1-mediated mitophagy by targeting GJA1 in myocardial IRI (Yan et al., 2023). However, more studies support that activation of mitophagy is protective and suggest that the phosphorylation state of FUNDC1 at Tyr18 may serve an essential role for several main reasons: first, under normoxic/physiological conditions, FUNDC1 phosphorylation level at Tyr18 is high and only slight phosphorylation occurs at Ser13, suggesting that Tyr18 phosphorylation mainly controls the baseline activity of FUNDC1; second, during hypoxia or ischemia, the phosphorylation levels of total FUNDC1 decreased significantly, while the Ser13 phosphorylation level of FUNDC1 remained relatively unchanged, indicating that the activation of FUNDC1 was achieved through dephosphorylation of Tyr18; third, during the reperfusion phase after ischemia or hypoxia, the phosphorylated FUNDC1^Tyr18^ changed slightly during the reperfusion phase after ischemia or hypoxia, whereas phosphorylated FUNDC1^Ser13^ gradually increased, indicating that phosphorylation of Ser13 inactivated FUNDC1 after reperfusion; finally, in general, the total phosphorylated FUNDC1 at Tyr18 and Ser13 sites decreased during the ischemic phase and gradually increased during the reperfusion phase, which was associated with FUNDC1-dependent mitophagy activation state was negatively correlated (Zhou et al., 2017a; Zhou et al., 2017b; Zhang et al., 2017; Zhou et al., 2018; Zhang, 2021). Although hypoxia and exercise-induced hypoxia are different, the research related to hypoxia has certainly provided ideas for the study of exercise.

## 6 Conclusion

In summary, the mechanisms regulating mitophagy are not a few mutually independent signaling pathways, but a multi-level regulatory network involving many cellular and mitochondrial mechanisms (Zhou et al., 2020). As a recently discovered receptor mediating mitophagy, FUNDC1 has been shown to not only participate in maintaining mitochondrial morphology and function but also perform an essential role in regulating mitophagy and mitochondrial dynamics, and new evidence suggests that FUNDC1 levels and phosphorylation status are significantly related to the onset, development and even prognosis for a variety of diseases, including heart disease, which predicts that FUNDC1 and its mediated mitophagy have tremendous potential applications in the intervention or treatment of numerous human diseases (Zhang et al., 2021b; Zhang et al., 2021d; Qiu et al., 2021; Sun et al., 2021; Zhang, 2021; Liu et al., 2022; Mao et al., 2022). However, our understanding of FUNDC1 physiological functions is still limited, for example, are there other targets that control FUNDC1-mediated mitophagy? How are the various regulatory targets activated or inhibited? Are there interactions, antagonisms, or compensatory effects between regulatory targets? FUNDC1 is also involved in the formation of MAMs, consistent with or contradictory to its role in mediating mitophagy and mitochondrial dynamics? Therefore, more comprehensive and in-depth studies are necessary. On the one hand, the enigmatic role of FUNDC1 in regulating mitochondrial dynamics and mitophagy remains incompletely elucidated. On the other hand, the mechanism behind the action of FUNDC1 in different diseases may be different, and new studies are urgently needed to give further plausible explanations.
